# Hospital Reform

**Published:** 1875-02

**Authors:** 


### HOSPITAL REFORM.

A mob entered an hospital full of yellow fever patients one rainy night, took them all out into a grassy field, and burned the building to ashes; and this within a few years and within a short distance of the City Hall of New York. No appreciable ill result followed in any single case.

Multitudes of cases were recorded during the war, showing that men left on the battle-field or placed in tents, more certainly recovered than those who were sent into the hospitals protected from rains and storms and night air.

It is known to every observant person who has visited hospitals, that there is a peculiar heaviness in the air, and a peculiar odor, however faultlessly clean the floor is and every observable nook and corner. It is just as well known that there is no more important element in the cure of disease than pure out-door air. It would seem, therefore, that hospital accommodations should consist of detached one-story buildings, with a front and rear room, the apartment to be occupied by the patient to have a window and a door facing the south, without any cellar beneath; the timbers on which the floor is laid to be fifteen inches thick and filled in with charcoal, or ashes, or sand, so as to prevent emanations from the earth reaching the apartment. There should be a few inches of space between the earth and the timbers, and openings left for the fresh air to come under the floors. Each apartment should have an open fire-place.

